# Effects of adding atracurium to Lidocaine solution during intravenous regional anesthesia in dogs

**DOI:** 10.1186/s13620-025-00303-5

**Published:** 2025-08-06

**Authors:** Marwa Abass, Alshimaa M. Farag, Reham A. Fahmy

**Affiliations:** 1 Department of Surgery, Anesthesiology, and Radiology, Faculty of Veterinary Medicine, Mansoura University, Mansoura, 35516 Egypt; 2 Department of Internal Medicine, and Infectious Diseases, Faculty of Veterinary Medicine, Mansoura University, Mansoura, 35516 Egypt; 3 Fellow of Veterinary Surgery, Oncology Centre, Mansoura University, Mansoura, 35516 Egypt

**Keywords:** Lidocaine, Atracurium, Dogs, Intravenous regional anesthesia, Nociception, Acute pain

## Abstract

**Background:**

Acute pain management and the efficacy of analgesic therapies are essential in orthopedic surgery on the distal extremities of dogs’ forelimbs. This is due to the manipulation of both soft and orthopedic tissues. Therefore, this study aimed to compare the antinociceptive, akinesia, cardiovascular, and plasma-level effects of adding atracurium to intravenous regional anesthesia (IVRA) with lidocaine in dogs.

**Methods:**

Fifty male Mongrel dogs weighing 15 ± 5 kg and aged 2.5 ± 0.6 years were premedicated with 0.045 mg/kg of atropine sulfate and 0.05 mg/kg of acepromazine. While under general anaesthesia, the dogs were randomly allocated into two IVRA groups (*n* = 25/group): the lidocaine group (LG; 3 mg/kg) and the atracurium (0.3 mg/kg) combined with the lidocaine (3 mg/kg) group (LAG). Following IVRA injections, the toe pinch response and nerve stimulation test were performed, with the contralateral limb serving as its control limb. The mean blood pressure (MAP), pulse rate (PR), respiratory rate (RR), end-tidal carbon dioxide level (EtCO_2_), rectal temperature, echocardiographic indices, and plasma lidocaine concentrations were measured.

**Results:**

At 25, 35, 45, and 55 min post-induction, the LAG exhibited a significantly lower (*P* ≤ 0.01) nociception limb withdrawal reflex score indicated by an absence of the limb withdrawal reflex (score 1) than the LG, which showed a mild limb trembling (score 2). Moreover, at 30, 40, 50, and 60 min post-induction, the LAG had an absence of the carpus twitch (score 1) with a significantly deeper degree of nerve block (*P* ≤ 0.01) compared to the LG. There were no significant differences in the physiological parameters between groups during anesthesia time. Meanwhile, the MAP, PR, and RR were significantly higher (*P* ≤ 0.05) in the LG than in the LAG post-nociception stimuli and during the recovery period. After tourniquet removal, hypersalivation and muscle tremors were observed in four dogs in the LAG and one in the LG.

**Conclusion:**

The use of IVRA with atracurium/lidocaine is a potentially effective IVRA agent for enhancing analgesia and akinesia in the distal extremities of dogs. However, it is important to consider the potential signs compatible with systemic toxicity that may occur, such as hypersalivation and muscle tremors, after releasing the tourniquet.

**Supplementary Information:**

The online version contains supplementary material available at 10.1186/s13620-025-00303-5.

## Background

Local and regional analgesia techniques present significant advantages in animals under general anesthesia, not only because they allow the control of negative effects at the cardiorespiratory level and surgical stress but also because they decrease the requirement for inhalation agents and opioid consumption [1–4].

In veterinary medicine, intravenous regional anesthesia (IVRA) is frequently utilized in ruminants [5–7]. However, there is a scarcity of research investigating this technique’s application in small animals during amputation of a toe, fracture repair, arthrodesis, and wound closure [8–10].

It is used as part of a multimodal perioperative pain management protocol, under either light or deep sedation or general anesthesia, to control intraoperative nociception and minimize the alveolar concentration of inhalant anesthetics [11]. IVRA is predominantly used in the distal extremities of ruminants due to its reliability, relative simplicity, and consistency as an analgesic technique for selected surgical procedures [7]. It is also known as Bier’s block, a form of regional analgesia that involves intravenously injecting a local anesthetic agent below a tourniquet to provide anesthesia and relieve mild to moderate pain in the structures distal to the tourniquet [12].

Lidocaine is one of the most commonly used local anesthetics for IVRA. However, it has limitations in terms of postoperative analgesia due to its short duration of action [13]. Therefore, in humans, several adjuvant drugs, such as muscle relaxants, opioids, NSAIDs, and ketamine, are added to local anesthetics to enhance akinesia and accelerate the onset of anesthesia with minimal side effects [14–17].

A combination of local anesthetics and neuromuscular blocking drugs such as atracurium or pancuronium has the potential to accelerate the motor and sensory nerve block during IVRA in humans. This interaction results in an extended and more intense neuromuscular block, resulting in more profound muscle relaxation. Consequently, it can reduce intra- and post-operative pain and enhance overall body analgesia [18–21]. Until now, there have been no documented problems linked to the use of adjuvant neuromuscular blocking drugs in IVRA. However, using neuromuscular blocking agents alone is not advisable [22].

To our knowledge, no study has compared the anesthetic and cardiorespiratory effects of IVRA with lidocaine/atracurium in small animals. Therefore, this study aims to compare the antinociceptive, akinesia, cardiovascular, and plasma-level effects of adding atracurium to intravenous regional anesthesia (IVRA) with lidocaine in dogs. We hypothesized that applying IVRA with a combination of atracurium and lidocaine on the distal extremities of the thoracic limbs (under the carpal joint in dogs) would prolong the duration of IVRA post-tourniquet removal, increase muscle relaxation, and minimize cardiopulmonary complications compared to using IVRA with lidocaine alone.

## Materials and methods

### Animal ethics and consent

**Fig. 1:**
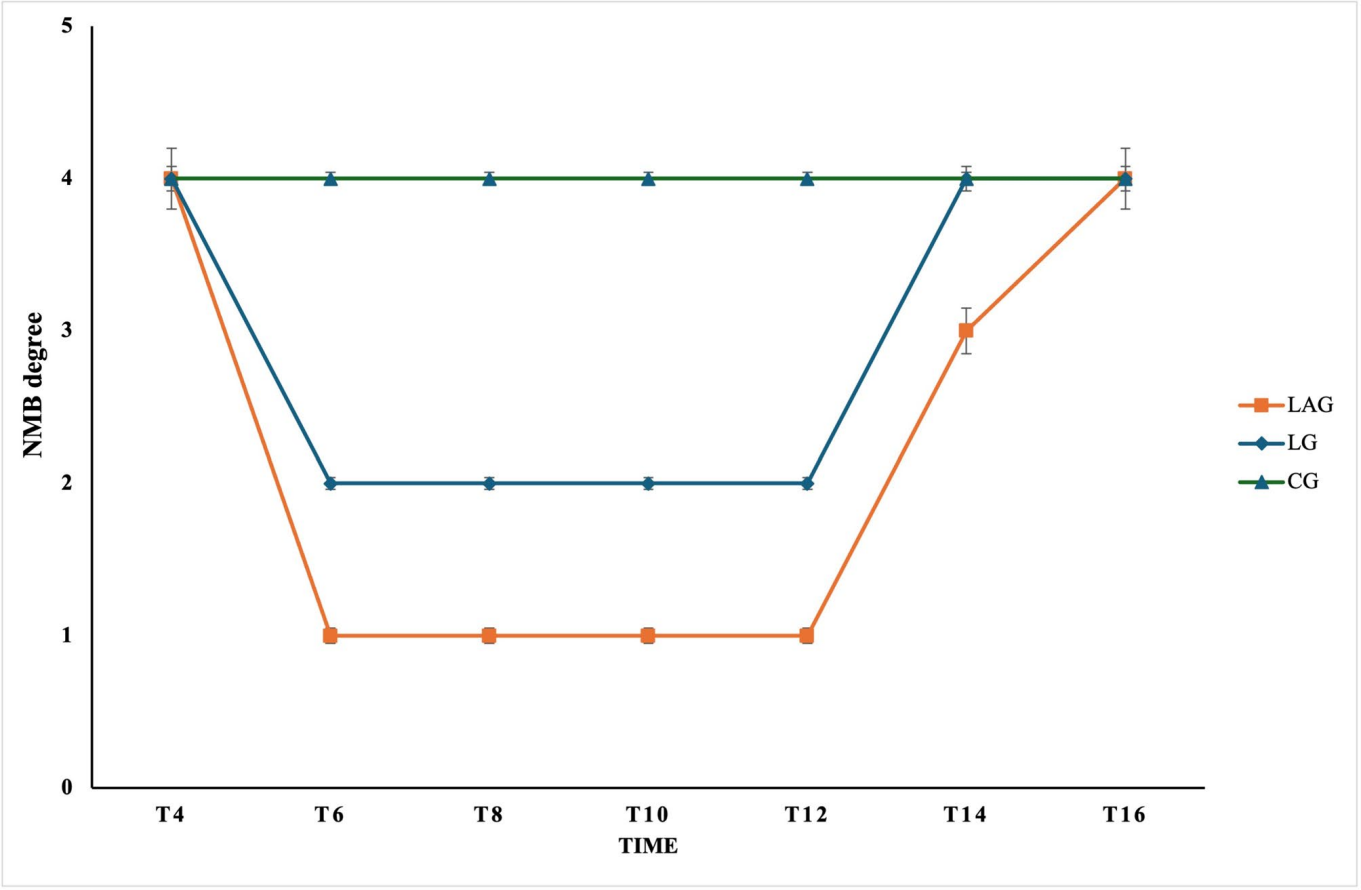
The schematic cartoon of the Methodology and the timeline of measurements. It was designed by the authors of the manuscript (Created with Biorender.com with permission)

All experiments were carried out following relevant guidelines and regulations. The Mansoura University Animal Care and Use Committee reviewed and approved this experiment, documented with code MU-ACUC (VM.R.23.11.130). All procedures were reported in the format specified by ARRIVE [23]. The experimental strategy is illustrated in the schematic cartoon (Fig. 1).

### Animals

A power analysis was conducted using G*Power version 3.1.9.7 to calculate the minimum sample size required based on the study hypothesis (a moderate level of muscle relaxation of 37.5%, a mild degree of nociception with minimal effect on CVS, and longer postoperative analgesia). A prospective sample size was calculated for the two groups. The sample size based on the Sen et al. [24] study, the sample size needed to achieve 90% power for detecting an effect at a significance level of α = 0.05, effect size d = 0.85, and the critical t = 1.689, was *N* = 50. The total sample size was set at 50 dogs (twenty-five per group).

A total of 50 male mongrel dogs, aged 2.5 ± 0.6 years and weighing 15 ± 5 kg, were utilized in the current study. The dogs were obtained from a shelter in Mansoura under the control of the Mansoura University-Animal Care and Use Committee. Before participating in the present study, the owner provided their informed consent. The eligibility and inclusion criteria for the current experiment were dogs with an American Society of Anesthesiologists (ASA) score of I; also, they were deemed healthy based on physical examination, complete blood count (CBC), and biochemistry tests. Dogs that had significant trauma or systemic disease, showed signs of nervousness or aggression, or had recently received drug therapy that could potentially impact sedative/anesthetic agents were excluded. Dogs completed 15 days of kennel acclimation before commencing the experimental treatment.

### Experimental design

#### Anaesthetic protocol

Before IV administration of the pre-anesthetic medications, food intake was restricted to 8 h, with free access to water. The anesthetic protocol was performed by an experienced anesthetist (RF). All dogs were premedicated with an intramuscular (IM) administration of 0.045 mg/kg of atropine sulfate (Atropine Sulphate, 1 mg/kg, MEMCO, Egypt) and 0.05 mg/kg of acepromazine (Prequillan, 10 mg/ml, Provet AG, Switzerland). Four peripheral intravenous catheters (PIVC, 20-gauge, Ultramed, Egypt) were placed, one in each limb. The catheters in the forelimbs were used for IVRA, while those in the hindlimbs were used for propofol infusion. Thirty minutes following premedication, each dog underwent an intravenous (IV) injection of 4 mg/kg propofol (Diprivan, 10 mg/kg, Sandoz, UK) for anesthetic induction. The calculated doses of propofol were slowly injected over 30 s until the endpoints were reached, indicated by the loss of medial palpebral reflex, pedal reflex, and jaw tone. If necessary, an additional induction dose of propofol (1 mg/kg) was administered. After the induction of anesthesia, the dogs were intubated and positioned in right lateral recumbency.

The propofol infusion rate was adjusted to 0.3 mg/kg/min [25, 26] to maintain a light plane of surgical anesthesia [9]. This was determined by recording a positive response in the toe pinch reflex of the right thoracic limb and the position of the eyeball at the medial canthus of the eye or nystagmus. If any of these reflexes were observed, the propofol infusion rate was adjusted to 0.4 mg/kg/min. Otherwise, the propofol titration rate was set at 0.3 mg/kg/min.

Each dog was under general anesthesia with propofol for one hour. If the dog did not initiate respiration within 30 s after induction, manual ventilation was provided by administering a positive pressure breath to 12–15 cmH_2_O using the rebreathing bag every 15 s until spontaneous respiration resumed. Ten minutes after induction, the thoracic limbs were lifted for five minutes to exsanguinate. Afterward, a compressive bandage was carefully wrapped around each limb, starting from the toe and extending to the middle of the humerus without disrupting the intravenous catheter.

A 6.5 cm-wide cuff connected to a sphygmomanometer (Riester Tourniquet 5255, Germany) was used as a pneumatic tourniquet and placed above the elbow joint; then the adhesive tape (SilkPlast adhesive tape, 10 cm, Pharmaplast, Egypt) was applied around it. The pressure of the pneumatic tourniquet cuff was adjusted to approximately 170 mmHg, above the mean blood pressure of the dogs. Then, the compressive bandage was removed, and a pulse oximeter probe (Basic veterinary monitor, Mindray, China) was attached to the interdigital area of the left thoracic limb. The absence of a signal indicates no blood flow in the limb.

Twenty minutes after the induction of anesthesia, the dogs were randomly (sealed envelope) assigned to one of the following treatment groups (*n* = 25 dogs/group) according to IVRA agents.

The lidocaine group (LG) received an IVRA of 3 mg/kg of lidocaine hydrochloride (Debocaine, 20 mg/mL, Adwia, Egypt). The lidocaine/atracurium group (LAG) received an IVRA of 3 mg/kg of lidocaine hydrochloride combined with 0.3 mg/kg of atracurium (atracurium hamein, 10 mg/mL, Hamein Pharma, Germany). The final volume of the IVRA treatments was adjusted to 0.6 mL/kg using normal saline and then injected into the left cephalic vein over five minutes.

The right thoracic limb of the dogs, considered the control group (CG), received an equal volume of normal saline solution (Sodium Chloride 0.9% solution, 500 ml, U Pharma, Egypt) injected into the right cephalic vein over five minutes. Following the completion of the IVRA volume injection, the extension IV lines in both thoracic limbs were withdrawn as a precautionary measure to avoid any inadvertent errors.

The tourniquets were maintained for 40 min (from T4 up to T12); after that, the tourniquets were deflated. There were four opening trials, each lasting 30 s with 5-minute intervals.

The deflation technique involved four opening trials, each lasting 30 s with 5-minute intervals at time points T12, T13, T14, and T15, before the cuffs were completely deflated and removed at time point T15. After detecting the swallowing reflex and tongue movement, the trachea was extubated. After complete recovery, all dogs received ketoprofen at a dose of 1.1 mg/kg intramuscularly (IM) and a prophylactic dose of cefazolin (22 mg/kg) every 12 h for three days.

#### Experimental evaluations

A General anaesthesia evaluation
  The induction time (minutes), recovery time (minutes), the increment dose of propofol (mg/kg), and the cuff pressure measured in each dog used during anesthesia were recorded.
B Intravenous Regional Anesthesia Evaluation
  The IVRA evaluation methods were performed by a single-blind observer unaware of the IVRA treatments (MA). The timetable and its indication for the current experiment evaluation are displayed in Table 1.
  i. Toe Pinch Withdrawal Response (TP)
    The first ratchet of a Halsted mosquito hemostat (Straight, 127 mm Miltex, Germany) was closed for two seconds to squeeze the second toes on both the control (right) and the test (left) thoracic limbs at each time point T4, T5, T7, T9, T11, T13, T15, and T16. The responses were scaled from no response, which scored 1 (indicating no nociception), to limb withdrawal (LWR), which scored 4 (indicating severe nociception), as shown in Table 2.
    The duration of postoperative analgesia was recorded from the moment the tourniquets were deflated at T12 until the end of the experiment at T16 (marked by the return of flexion and limb withdrawal) [6, 9, 27].
  ii. Nerve Stimulation Test (NST)
    The NST was used to monitor neuromuscular function by measuring carpal joint twitch responses to a supramaximal electrical stimulus applied to the ulnar nerve. A peripheral nerve stimulator (Myotest, Biometer International, Odense, Denmark) was used to perform a train of four stimuli (TOF). It consisted of four supramaximal stimuli of 200 ms at a frequency of 2 Hz and an intensity of 60 mA. An ulnar nerve stimulating electrode was placed above the site of the ulnar nerve on the lateral side of the flexor carpi ulnaris muscle tendon, 1 cm from the accessory carpal pad [28, 29]. This stimulus was repeated at regular 10-minute intervals following the IVRA injection. Neuromuscular stimulation (NST) was applied on both forelimbs at T4, T6, T8, T10, T12, T14, and T16 [30]. This arrangement stimulates the flexor carpi ulnaris muscle that flexes the carpus, as depicted in Table3. The carpal flexion was recorded, and the disappearance of the response to all four stimuli indicated a complete neuromuscular block [31].
  iii. Physiological parameters
    The physiological parameters included the end-tidal carbon dioxide level (EtCO_2_; mm Hg), pulse rate (PR; bpm), respiratory rate (RR; bpm), mean arterial blood pressure (MAP; mmHg), and rectal temperature (RT; ^o^C) were monitored via an animal multi-parameter veterinary monitor (MSLCW01, USA) at T0, T1, T3, T4, T5, T6, T7, T8, T9, T10, T11, T12, T13, T14, T15, and T16.
  iv. Echocardiographic parameters
    The area between the 4^th^ and 6^th^ intercostal spaces was prepared for each dog. After clipping the hair and applying acoustic gel for ultrasonographic measurements, the dogs were positioned in the right lateral recumbence. The echocardiographic images of each dog were recorded using a 5.0 MHz micro-convex transducer in the two-dimensional guided M-mode in the short-axis right parasternal plane echocardiography (SIUI, CTS-900V). The recordings were taken at four different time points: T0, T3, T6, and T16. Measurements were conducted to determine the internal diameter of the cardiac chambers and the thickness of their walls [32].
    The left ventricular end-diastolic diameter (LVDD mm) was determined at the point of maximum diameter, and the left ventricular systolic diameter (LVSD mm) was noted at the peak upward deflection of the caudal (posterior) wall. The posterior wall thickness was detected at end-systole. An echocardiographic investigation measured Stroke volume (SV ml), fractional shortening (FS%), and ejection fraction (EF%), which were calculated using ultrasonographic echocardiography software.
  v. Plasma Lidocaine Concentration
    The vein blood samples (3 ml) were collected from the cephalic vein of the right thoracic limb in heparin tubes and then were centrifuged at 1300 × *g* and 4°C for 10 minutes at each time point T5, T7, T9, T11, T13, T15, and T16. Lidocaine plasma concentrations (μg/mL) were tested in both groups using a gas chromatography technique with a nitrogen-selective sensor. The quantities limit of detection of the assay was 50 ng/mL. The evaluation times for lidocaine and the internal standard were around 5.1 and 9.1 minutes, respectively [33].

**Table 1:** Timetable and its indication for the current experimental evaluation

Time	Indication
T0	Before premedication (baseline value)
T1	Before induction
T2	10 min post-induction
T3	15 min post-induction
T4	20 min post-induction
T5	25 min post-induction
T6	30 min post-induction
T7	35 min post-induction
T8	40 min post-induction
T9	45 min post-induction
T10	50 min post-induction
T11	55 min post-induction
T12	60 min post-induction
T13	5 min post-end of propofol titration
T14	10 min post-end of propofol titration
T15	15 min post-end of propofol titration
T16	20 min post-end of propofol titration

**Table 2:** The limb withdrawal reflex (LWR) scores after toe pinch (TP) in dogs

The severity of nociception	Dog response	Score
Severe	Marked flexion and limb withdrawal	4
Moderate	Slight limb withdrawal	3
Mild	Limb trembling	2
Absent	No response	1

**Table 3:** The train-of-four (TOF) stimulation response in dogs

Approximate (TOF) ratio%	Animal Response (carpus twitch)	Score	NMB Degree
90%−100%	Cannot be determined	4	Normal function
40–80%	TOF count 4 without fade.	3	Minimal
39%	More than one twitch of the carpus	2	Moderate
0%	Absent of the twitch of the carpus	1	Deep (complete NMB)

### Statistical analysis

The data statistical analysis was conducted using SPSS software (version 29, IBM Support, USA). A Kolmogorov-Smirnov test was used to examine the data’s normal distribution. The parametric data were represented as mean ± standard deviation (SD), and the non-parametric data were represented as median (min-max).

Linear mixed effect models were used to compare the response to toe pinch withdrawal response, NMB, the physiological variables (EtCO2, PR, RR, MAP, and RT), echocardiographic parameters, and plasma concentration of lidocaine with the fixed effects of treatment, time, and the interaction of treatment and time. Additionally, to account for the repeated measurements, random effects of each dog and the interaction of dog and treatment were included in the model. The significant threshold was set ≤ 0.05.

## Results

### General anesthesia

The duration of analgesia of IVRA post deflation of tourniquets was significantly longer (*P* = 0.0481) in the LAG (20 min) versus the LG (15 min).

No complications occurred during the induction of anesthesia, catheterization, tracheal intubation, maintenance of anesthesia, and IVRA technique. During the recovery period, the deflation of tourniquets at T14, muscle tremors (involuntary, rhythmic, and oscillatory movements of a body part), and hypersalivation (inability to retain saliva in the oral cavity) were observed in one dog in the LG for 2 min and in four dogs in the LAG for 3.2 ± 0.91 min. The dogs uneventfully recovered, and all dogs were injected with one dose of IVRA during the experiment.

### Toe pinch withdrawal response (TP)

In general, the nociception scores of TP in the left limbs (tested limbs) were significantly lower in the LAG compared to the LG at T5, T7, T9, T11, T13, and T15 (*P* ≤ 0.01).

However, at T5, T7, T9, and T11, the nociception score of the left limbs (testing limbs) of the LG was 2, which showed a significant difference compared to the right control limbs (*P* ≤ 0.025). Meanwhile, the LAG scored 1, showing a significant decrease compared to the control limbs (*P* ≤ 0.008) and the LG group (*P* ≤ 0.01).

**Fig. 2:**
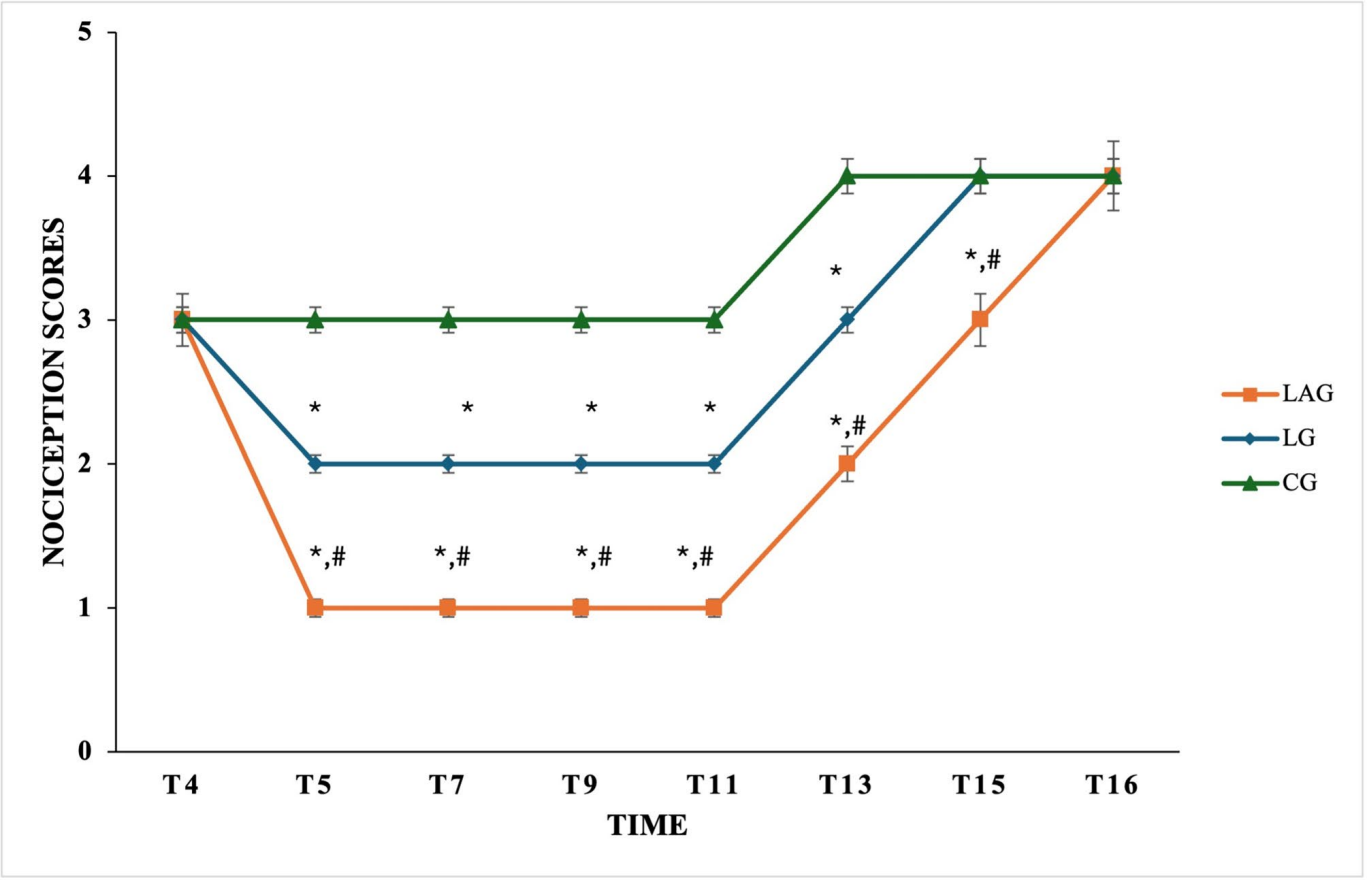
Nociception scores post intravenous regional analgesia (IVRA) injection with the lidocaine group (LG), the lidocaine/atracurium group (LAG), and the control group (CG) in anesthetized dogs at T4, T5, T7, T9, T11, T13, T15, and T16

The scores of TP gradually increased until complete recovery at T13, T15, and T16 in both groups, which scored 3, 4, and 4 in the LG versus 2, 3, and 4 in the LAG (Fig. 2).

### Nerve stimulation test (NST)

At T6, T8, T10, T12, and T14, the TOF ratio was significantly higher in the LG (*P* ≤ 0.01) compared to the LAG. However, at the same measured times, both groups were substantially lower in the TOF ratio than the CG (*P* ≤ 0.03).

The TOF ratio in the LG was 39%, indicating a moderate degree of NMB (score 2). In comparison, the LAG had a deep level of the TOF ratio of 0%, indicating a deep degree of NMB (score 1), while in the CG, the TOF ratio exhibited 97%, indicating normal nerve function (score 4) at T6, T8, T10, and T12.

**Fig. 3:**
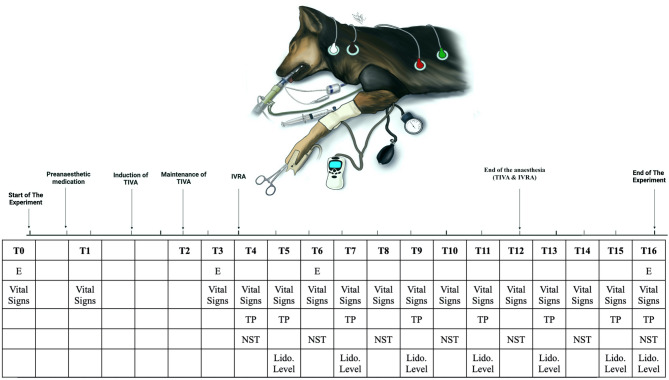
Neuromuscular block (NMB) score after intravenous regional analgesia (IVRA) injection with the lidocaine group (LG), the lidocaine/atracurium group (LAG), and the control group (CG) in anesthetized dogs at T4, T6, T8, T10, T112, T14, and T16

At T14 and T16, there was no significant difference in the TOF ratio between the LG and the CG, both showing 98% (score 4). However, in the LAG, the TOF ratio at T14 was 45%, indicating a minimal degree of NMB (score 3). At T16, the TOF ratio was 100% associated with a complete restoration of the nerve response (Fig. 3).

### Physiological parameters

During the study period, the physiological parameters, including EtCO_2,_ PR, RR, and MAP, were statistically decreased over time within the same group at different measuring time points (T1, T3, T4, T5, T6, T7, T8, T9, T10, T11, and T12) compared to the baseline time (T0) (*P* ≤ 0.045).

However, the PR, RR, and MAP values suddenly increased significantly (*P* ≤ 0.021) in the LG compared to the LAG at T5, T7, T9, and T11. Whereas the PR, RR, and MAP values at T13, T14, and T15 were sustainably higher (*P* ≤ 0.05) in the LG compared to the LAG, as shown in Table 4.

**Table 4:** Changes in (PR; bpm), respiratory rate (RR; bpm), mean blood pressure (MAP; mmHg), and rectal temperature (RT; ^o^C), between the lidocaine group (LG) and atracurium/lidocaine (LAG) in dogs at baseline T0; before induction (T1), and at T3, T4, T5, T6, T7, T8, T9, T10, T11, T12, T13, T14, T15, and T16

Time	Groups	EtCO_2_ (mmHg)	PR (bpm)	RR (bpm)	MAP (mmHg)	RT (^o^C)
T0	LG	38.5 ± 1.9	120 ± 4.6	23.1 ± 1.9	89 ± 8.0	38.1 ± 0.4
	LAG	38.7 ± 2.4	119 ± 2.2	23.5 ± 3.1	90 ± 4.0	38.3 ± 0.3
T1	LG	38.6 ± 2.1	99 ± 1.4^*^	18.2 ± 0.16^*^	83 ± 6.2^*^	37.4 ± 0.2
	LAG	38.6 ± 1.1	97 ± 2.8^*^	17.7 ± 1.02^*^	85 ± 2.8^*^	37.5 ± 0.2
T3	LG	40.1 ± 3.03^*^	96 ± 0.94^*^	17.8 ± 1.23^*^	82.8 ± 5.1^*^	37.5 ± 0.2
	LAG	40.4 ± 1.8^*^	99 ± 2.46^*^	17.2 ± 2.82^*^	84 ± 4.7^*^	37.6 ± 0.1
T4	LG	40.4 ± 1.2^*^	96.3 ± 2.32^*^	17.3 ± 3.24^*^	82.5 ± 4.5^*^	37.5 ± 0.2
	LAG	40.7 ± 1.1^*^	98.8 ± 3.15^*^	17 ± 3.51^*^	83.6 ± 3.8^*^	37.6 ± 0.1
T5	LG	40.7 ± 2.32^*^	108.9 ± 3.12^*,a^	18.5 ± 2.16^*,a^	86.2 ± 5.2^*,a^	37.6 ± 0.3
	LAG	40.9 ± 2.03^*^	100 ± 2.67^*,b^	17 ± 2.94^*,b^	84 ± 3.6^*,b^	37.7 ± 0.6
T6	LG	40.8 ± 1.5^*^	98.4 ± 2.93^*^	17.1 ± 0.98^*^	83.5 ± 2.1^*^	37.9 ± 0.1
	LAG	40.6 ± 2.3^*^	97 ± 3.01^*^	16.5 ± 2.60^*^	82.8 ± 3.2^*^	37.8 ± 0.5
T7	LG	40.4 ± 2.1^*^	110.3 ± 3.82^*,a^	18.7 ± 4.53^*,a^	86 ± 3.76^*,a^	37.8 ± 0.74
	LAG	40.5 ± 1.5^*^	101.9 ± 2.74^*,b^	17.3 ± 3.62^*,b^	84.1 ± 2.18^*,b^	37.7 ± 0.33
T8	LG	41.1 ± 1.8^*^	98.6 ± 5.14^*^	17.7 ± 2.40^*^	83.7 ± 2.1^*^	37.9 ± 0.20
	LAG	41.6 ± 2.1^*^	97.1 ± 1.60^*^	16.9 ± 4.11^*^	82.1 ± 3.35^*^	38 ± 0.33
T9	LG	41.1 ± 1.8^*^	110.6 ± 5.14^*,a^	18.6 ± 2.40^*,a^	86.2 ± 2.65^*,a^	37.9 ± 0.20
	LAG	41.6 ± 2.1^*^	99.51 ± 1.60^*,b^	17.1 ± 4.11^*,b^	84.3 ± 1.42^*,b^	38 ± 0.33
T10	LG	41.2 ± 2.1^*^	98.7 ± 3.65^*^	17.4 ± 2.10^*^	83.5 ± 3.11^*^	37.8 ± 0.43
	LAG	41.54 ± 1.1^*^	97.2 ± 2.51^*^	16.8 ± 3.01^*^	82.2 ± 4.30^*^	38.1 ± 0.40
T11	LG	40.2 ± 3.1^*^	111.5 ± 2.32^*,a^	18.6 ± 2.61^*,a^	86.2 ± 2.07^*,a^	37.9 ± 0.4
	LAG	40.7 ± 2.5^*^	102 ± 3.15^*,b^	17.4 ± 4.01^*,b^	84.2 ± 3.12^*,b^	37.9 ± 0.3
T12	LG	41.3 ± 1.3^*^	95 ± 3.24^*^	16.5 ± 5.7^*^	83.7 ± 2.1^*^	37.8 ± 0.4
	LAG	41.5 ± 1.2^*^	95 ± 1.61^*^	16.7 ± 4.6^*^	83 ± 2.3^*^	37.7 ± 0.3
T13	LG	41.5 ± 1.3^*^	115 ± 0.30^*,^	19.6 ± 2.83^*, a^	88 ± 1.94^*, a^	37.8 ± 0.4
	LAG	41.6 ± 0.92^*^	98 ± 0.36^*, b^	17.3 ± 3.23^*, b^	83 ± 4.3^*, b^	37.7 ± 0.3
T14	LG	41.6 ± 2.4^*^	123 ± 1.50^*, a^	21.2 ± 2.06^*,a^	95 ± 3.61^*^	37.8 ± 0.4
	LAG	41.7 ± 2.2^*^	100 ± 1.36^*,b^	19.1 ± 1.92^*,b^	89 ± 2.62^*^	37.7 ± 0.3
T15	LG	41.4 ± 2.4^*^	128 ± 2.04^*, a^	22.4 ± 1.91^*, a^	98 ± 1.9^*, a^	38.1 ± 0.1
	LAG	41.8 ± 2.2^*^	118 ± 0.36^*,b^	20.3 ± 2.13^*, b^	91.2 ± 4.3^*, b^	38.1 ± 0.2
T16	LG	40.4 ± 1.1^*^	131 ± 0.84^*^	25.3 ± 0.36^*^	95 ± 8.0^*^	38 ± 0.04
	LAG	42 ± 1.35^*^	128 ± 1.6^*^	25.7 ± 0.21^*^	93.3 ± 2.32^*^	38 ± 0.93

* Significant difference between baseline T0 and define time *P* ≤ 0.05

^a, b^ Letters significant difference between groups at the same time *P* ≤ 0.05

### Echocardiographic parameters

The echocardiograph images showed no significant difference between the groups (*P* ≥ 0.561) at baseline (T0) before preanesthetic medication. However, the echocardiograph profiles T3, T6, and T16 had significantly lower values (*P* ≤ 0.05) than baseline T0. Additionally, the LAG had notably lower values (*P* ≤ 0.05) at T16 compared to the LG Table 5.

**Table 5:** Changes in the left ventricular end-diastolic diameter (LVDd; mm), the left ventricular systolic diameter (LVDs; mm), stroke volume (SV ml), fractional shortening (FS%), and ejection fraction (EF%) between the Lidocaine group (LG) and atracurium/lidocaine (LAG) in dogs at baseline T0; before induction (T1), and at T3, T6, and T16

Time	Group	LVDd (mm)	LVDs (mm)	SV (ml)	FS %	EF%
T0	LG	45.63 ± 2.78	30.41 ± 2.35	18.6 ± 2.16	45 ± 3.62	65.8 ± 5.3
	LAG	46.28 ± 1.21	31.33 ± 5.11	18.21 ± 2.5	44 ± 5.33	65.8 ± 5.3
T3	LG	43.13 ± 1.91*	28.45 ± 5.41*	17.04 ± 1.43*	43 ± 3.12^*^	64 ± 4.1^*^
	LAG	42.33 ± 2.65*	29.17 ± 4.12*	16.92 ± 2.61*	43 ± 3.12^*^	63.7 ± 4.1^*^
T6	LG	42.98 ± 2.19*	27.50 ± 6.31*	16.82 ± 3.37*	43 ± 2.25^*^	63.3 ± 4.7^*^
	LAG	41.95 ± 5.24*	27.32 ± 2.98*	16.59 ± 4.22*	42 ± 2.25^*^	63.2 ± 2.58^*^
T16	LG	40.17 ± 2.04*	27.82 ± 3.68^*^	16.5 ± 3.03^*^	42 ± 1.54^*^	61.3 ± 6.12^*^
	LAG	39.17 ± 3.64^*, b^	25.09 ± 4.73^*, b^	15.91 ± 1.32^*, b^	39 ± 1.54^*, b^	59.93 ± 3.41^*, b^

* Significant difference between baselineT0 and define time *P* ≤ 0.05

^a, b^ Letters significant difference between groups at the same time *P* ≤ 0.05

### Plasma Lidocaine concentration

**Fig. 4:**
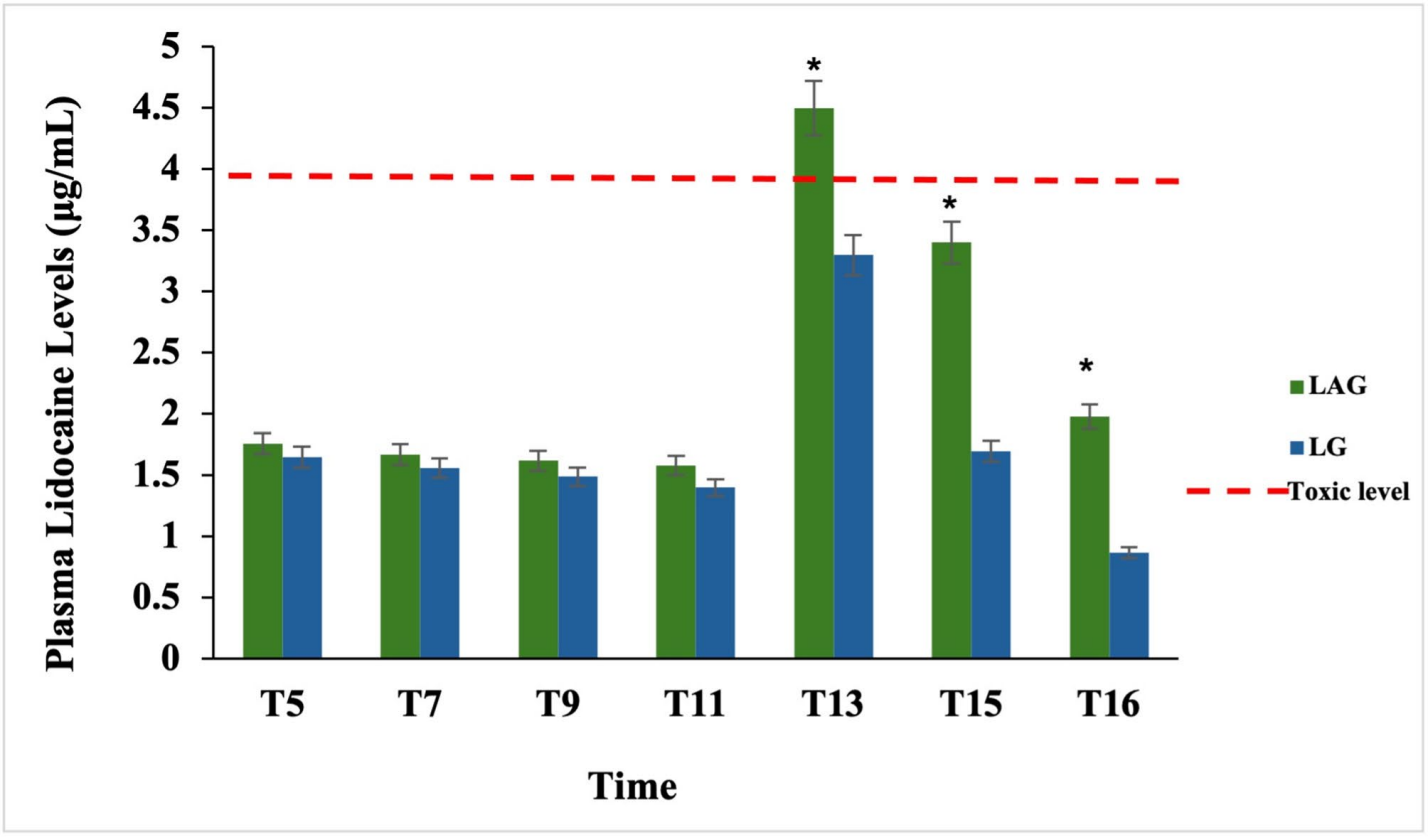
Plasma level concentration of lidocaine (mcg/ml) between the lidocaine group (LG) and atracurium/lidocaine (LAG) in dogs at T5, T7, T9, T11, T113, T15, and T16

There were no statistical differences between both groups at T5, T7, T9, and T11. The plasma concentrations of lidocaine in LAG were 1.8 ± 0.3, 1.7 ± 0.23, 1.6 ± 0.7, and 1.6 ± 0.5 µg/mL whereas in LG were 1.65 ± 0.13, 1.6 ± 0.2, 1.5 ± 0.4, and 1.4 ± 0.1 µg/mL, respectively. The plasma concentrations of lidocaine were significantly higher (*P* < 0.01) in the LAG than in the LG at T13, T15, and T16. Specifically, plasma lidocaine concentrations in the LAG were 4.5 ± 1.3, 3.4 ± 3.1, and 1.98 ± 2.9 µg/mL; in comparison, the LG had plasma lidocaine concentrations of 3.3 ± 3.3, 1.7 ± 2.2, and 0.87 ± 1.83 µg/mL. Plasma lidocaine concentrations in the LAG had a prolonged duration compared to those in the LG (Fig. 4).

## Discussion

The most significant finding of the current experiment was that adding atracurium to lidocaine during IVRA in dogs improved antinociceptive criteria and akinesia effectiveness. IVRA is a safe and cost-efficient method for providing short-term anesthesia and decreased nociception during surgery on extremities (thoracic and pelvic limbs), especially in severely compromised cases. However, it is crucial to consider that there is a rapid loss of anesthesia and a return of sensation after deflating the tourniquet [27, 34–36].

Lidocaine is commonly used during IVRA in veterinary medicine. In cats and dogs, a dose of 3 mg/kg of lidocaine 0.5–2% is typically administered during IVRA, with a final volume of treatment being 0.6 mL/kg [8, 10, 27, 37]. In our experiment, lidocaine was used at a dose of 3 mg/kg and a final volume of 0.6 mL/kg (i.e., 0.4%) as suggested [10]. Likewise, previous studies conducted on humans and dogs have shown that high doses of lidocaine during IVRA can lead to life-threatening side effects [8–10, 37, 38]. Webb et al. [8] declared that tachycardia was recorded in dogs undergoing finger amputation despite the administration of IVRA with lidocaine.

There are synergistic effects between lidocaine and other drugs, such as neuromuscular blockers, dexmedetomidine, ketamine, and paracetamol, during IVRA in humans [21, 39–41]. The use of atracurium as an adjuvant in IVRA is due to its effect on muscle spindles: It reduces central input from these structures, which results in loss of muscle tone and control of voluntary movements, with a decrease in nervous inputs to the brain, so that facilitate fracture reduction, reduce pain during and after the procedure, and may obviate the need for general anesthesia and hospital admission [42].

Due to the lack of information regarding the use of atracurium and lidocaine during IVRA in veterinary medicine, the present study compared the impact of administering IVRA of lidocaine alone versus a combination of lidocaine and atracurium by measuring sensory and motor block, changes in physiological parameters, and echocardiographic variables. Based on the results of our pilot study (unpublished data), it was found that IVRA of atracurium alone was not effective in inhibiting the TP or NST. It could be attributed to the low dosage used in this experiment (0.3 mg/kg). Toshitsugu et al. [43] mentioned that doses of atracurium that may produce complete NMB in healthy goats during general anesthesia were 0.5–0.75 mg/kg.

The effective use of tourniquets before IVRA injections aims to increase the concentration and effectiveness of IVRA agents, as well as to prevent the dilution or leakage of these agents under the tourniquet. As a result, the risk of drug toxicity can be minimized by avoiding the release of a huge volume of blood-containing treatments into circulation [9, 10, 34].

Previous studies [44–47] had recorded signs of local anesthetics toxicity following IVRA with lidocaine in dogs, including severe tachycardia, apnea, hypersalivation, seizures, muscle tremors, and sedation. Unlike our results, these signs were not observed during proper inflation (170 mm Hg) of the tourniquets from T4 up to T12 (i.e., 0–40 min post-IVRA).

This result aligns with the findings of De Marzo et al. [37] during pancarpal arthrodesis in anesthetized dogs undergoing IVRA. This finding may be attributed to the effective use of tourniquets before and during IVRA injections, in addition to following the necessary precautions. These precautions involve elevating the tested limb for five minutes, applying a compressive bandage for proper exsanguination, using a pneumatic cuff, maintaining a tourniquet pressure that is at least 170 mmHg above the mean blood pressure, monitoring pulse oximeter measurements, and ensuring the absence of an arterial pulse at the tested limb [10, 34, 48]. Avoid excessive and unnecessary pressure on the tourniquet, which causes mild to moderate pain and ischemia [9, 10]. Lastly, the IVRA treatments were injected over five minutes to avoid excessive pressure [37, 49–51]. Therefore, in the present study, to ensure adequate closure of arteries, the cuff width of the pneumatic tourniquet was 6.5 cm, which exceeded 20% of the diameter of the limbs of dogs. Additionally, the pneumatic tourniquet cuffs were inflated up to 170 mm Hg, which was higher than the MAP of the anesthetized dogs.

Moreover, gradually deflating the tourniquets was a vital step after the end of IVRA to avoid the sudden release of a high concentration of treatments into the bloodstream and toxicity. Imani Rastabi et al. [9] recommended a 30-second opening time for a tourniquet within a 5-minute interval between more than three opening trials. In the present study, there were four opening trials, each lasting 30 s with 5-minute intervals to reduce the incidence of drug toxicity.

In our study, during the deflation of the tourniquets at T13, one dog in the LG group showed hypersalivation and muscle tremors for 2 min. After that, the dog recovered uneventfully. This result may be explained by a high level of plasma lidocaine (4.73 mcg/ml). Moreover, lidocaine interferes with the large transient sodium influx during membrane depolarization after the tourniquet was removed, as mentioned in previous studies in humans and dogs [9, 52].

On the same side, four dogs in the LAG group exhibited the above symptoms for 3.2 ± 0.91 min during the recovery period at T14, and the TOF ratio was 45%. This result could be explained by previous studies [53, 54], as muscle tremors and salivation post-IV injection of atracurium are due to residual atracurium in the intravenous fluid line and its low dose level in the bloodstream, called residual block or postanesthetic recurarization (side effect of NMB). Although TOF is in light of possible residual paralysis, concerning patient safety. Furthermore, acetylcholinesterase inhibitors and muscarinic effects of atracurium are the main causes of salivation and muscle tremors.

In the present study, the nociception severity following the TP in the LG was moderately characterized by a slight limb withdrawal with a score of 3 at T5. Whereas, at T7, T9, and T11 (i.e., 15, 25, and 35 min after the IVRA injection of lidocaine), the severity of nociception was mild, characterized by limb trembling with a score of 2. Additionally, the TOF% ratio was 39%, described by more than one twitch of the carpus at T6, T8, T10, and T12.

These results were consistent with those of Imani Rastabi et al. [9] and indicated inadequate NBM, unlike De Marzo et al. [37], who performed pan-arthrodesis in dogs, and Webb et al. [8], who amputated the fourth digit of a dog after a contaminated laceration using general inhalation anesthesia and IVRA with lidocaine 2%. This can be attributed to several reasons. Firstly, the administration of painkillers during the preanesthetic medication period. Secondly, deep surgical plane anesthesia was used. Thirdly, the utilization of a large volume and concentration of lidocaine. Lastly, there were variations in tissue viability, and the animals’ health status may also play a role.

Conversely, nociception was absent in the LAG with a score of 1 at T5, T7, T9, and T11, as well as TOF% was 0% described by complete NMB and absence of carpus twitch at T6, T8, T10, and T12 (i.e., lasting 40 min after IVRA injection of lidocaine/atracurium). The current study results are in agreement with previous human studies [15, 21, 39, 55] in which the addition of atracurium to lidocaine during IVRA improved the quality of IVRA, exhibited lower nociception scores, complete motor blockage [19, 56], and prolonged analgesic time after the tourniquet was released than IVRA with lidocaine alone. Furthermore, it leads to the diffusion and spreading of the IVRA agents to adjacent tissues and nerves [37, 57, 58].

The nerve function was fully restored rapidly, with a nociception score of 3, 4, and 4 in the LG at T13, T15, and T16, respectively. This rapid restoration of nerve function may be attributed to the rapid metabolism and short duration of lidocaine, as mentioned (11,47). In contrast, the nociception score in the LAG was moderate, with a score of 2 at T13 as well as the residual paralysis effect of atracurium.

The TOF method was used to detect significant and successful improvement in the motor block and evaluate the potential effects of IVRA agents [37, 59].

In the LAG, the skeletal muscle of the thoracic limb (flexor digitorum superficialis muscle) was completely or partially paralyzed due to the effects of atracurium action [60]. This rendered the limb withdrawal reflex (LWR) following the TP test inadequate and unreliable for detecting the degree of anti-nociception. Taking into account The Niyom’s study [61], which mentioned that the sensitivity of skeletal muscles to NMB depends on the types of muscle fibers, with type 2 muscle fibers being more resistant to non-depolarizing neuromuscular blocking drugs than type 1 muscle fibers, it is important to note that the most common muscle fiber type in the thoracic limb muscle (flexor digitorum superficialis muscle) is type 1. This suggests that the thoracic limb muscle may overestimate the residual neuromuscular blockade. Therefore, from all of the factors mentioned above, the variables in vital signs are the most important, reliable and accurate indicators to detect anti-nociception of the LAG.

The PR, RR, and MAP values were significantly higher after the TP at T5, T7, T9, T11, T13, and T15 in the LG than in the LAG. These results can be explained by Ruíz-López et al. [62] as changes in physiological variables, including heart rate, blood pressure, and respiration, which are important factors in actigraphy for measuring nociception in dogs and cats. This may suggest an increased level of nociception in the LG. However, at T16, there were no significant changes in the vital signs between groups, which may be due to the lack of systemic or local effects of IVRA. Consistent with the findings reported in a study on humans [63], there was no occurrence of apnea in either group following IVRA in the current experiment.

In our study, echocardiography results confirmed that IVRA agents had little effect on cardiac systolic function and left ventricle (LV) myocardial contractility during the study at T3 and T6 than baseline (T0). That was evidence of the successful application of tourniquets without leakage of IVRA agents. Unlike at T16 (after the complete removal of tourniquets), there were significant decreases (*P* = 0.023) in LVDd, LVDs, SV, EF%, and FE% in the LAG and the LA than base baseline (T0).

Lemo et al. [44] reported that the serum concentration of lidocaine that causes toxicity was 2.7 ± 1.3 µg/mL. Our results found that the peak plasma concentration of lidocaine was 4.5 ± 1.3 µg/mL in the LAG versus 3.3 ± 1.7 µg/mL in the LG at T13, which was the time at appeared the signs of toxicity appeared in five dogs (one in the LG and four in the LAG). These results were explained by Chadwick [64] as a high infusion rate of local anesthetic agents can result in high plasma concentrations and toxicity because of the lack of equilibration, which limits the redistribution of drugs and hepatic metabolism, even with low doses. Thus [9], it is recommended that shortening the opening times (than 30 s) and increasing the time interval between opening trials (than 5 min) during deflation of tourniquets may prevent toxicity. This study was limited to detecting atracurium concentrations in blood. However, previous studies [65, 66] have shown that plasma enzymes metabolize atracurium, whereas liver enzymes degrade lidocaine. Both drugs are known to be rapidly metabolized.

It was a worthy and important finding in the present study that the plasma concentration of lidocaine was notably higher than in the LG at T13, T15, and T16. This may be attributed to atracurium having a high affinity for plasma protein binding [67].

One of the main limitations of this study is the ability to differentiate between nociception and akinesia (paralysis). The current study tested the toe pinch as a measure of nociception, but it’s not clear if, at the same time, other parameters were evaluated (HR, RR, Blood pressure from baseline). The lack of response could have been just due to the presence of paralysis instead of a lack of nociception, and this is also addressed in the discussion when the absence of nociception matched the TOF 0%. Another crucial limitation of the present study was the inability to detect concentrations of atracurium or its metabolite concentrations during IVRA, as well as the lack of information to determine the impact of IVRA on volatile anesthesia, which is commonly used in small animal clinics. Another limitation of the current study was that it was conducted on healthy dogs with normal vital tissues. Diseased tissue has a different pH, which retard the action of the local anesthetics and leads to a different response, so clinical applications are recommended, such as orthopedic cases.

## Conclusions

It has been concluded that adding non-depolarizing neuromuscular blocking agents, such as atracurium, to local anesthesia, such as lidocaine, during IVRA can provide a satisfactory level of akinesia and anti-nociceptive effects with minimal alert on cardiopulmonary function in dogs. Moreover, it can prolong the nerve block duration and achieve a higher level of muscle relaxation. Therefore, this combination can be considered an effective locoregional method in healthy dogs, particularly when used with general anesthesia. Taking into account its negative effects on certain echocardiographic parameters such as reductions in LVD, SV, EF, and FE, as well as hypersalivation and tremors, it is recommended to release the tourniquet slowly. Caution should also be taken when using this combination in canine patients with cardiovascular disease.

## Supplementary Information

Supplementary Material 1.

Supplementary Material 2.

Supplementary Material 3.

Supplementary Material 4.

## Data Availability

Data is provided within the manuscript.
