# Supplementary material for: Effects of adding atracurium to Lidocaine solution during intravenous regional anesthesia in dogs
Source: Ir Vet J. 2025 Aug 6;78:16. doi: 10.1186/s13620-025-00303-5 (PMC12330004; doi:10.1186/s13620-025-00303-5)
Supplement: Supplementary file 1 — Supplementary Material 1. [file 13620_2025_303_MOESM1_ESM.pdf]

CERTIFICATE OF ENGLISH EDITING

THIS DOCUMENT CONFIRMS THAT THE MANUSCRIPT LISTED BELOW WAS EDITED BY AN ENGLISH LANGUAGE EDITOR WHO HOLDS A MASTER'S DEGREE IN APPLIED LINGUISTICS AND WORKS AS AN ASSISTANT LECTURER IN THE DEPARTMENT OF APPLIED LINGUISTICS AND INSTRUCTION (TEFL), AIN SHAMS UNIVERSITY, AND PH. D CANDIDATE AT UNIVERSITY OF ALBERTA, CANADA.

I HEREBY CONFIRM THAT ALL LINGUISTIC PROBLEMS HAVE BEEN ADDRESSED, AS WELL AS THE FOLLOWING ISSUES HAVE BEEN CORRECTED: GRAMMAR, PUNCTUATION, SPELLING, SYNTAX, PHRASING, STYLE, AND SENTENCE STRUCTURE.

Manuscript Title

EFFECTS OF ADDING ATRACURIUM TO LIDOCAINE SOLUTION DURING INTRAVENOUS REGIONAL ANAESTHESIA IN DOGS

Authors

MARWA ABASS , SHIMZ FARAG & REHAM FAHMY

SIGNATURE

Marwa Abolfotouh

FOR CONTACT

abolfoto@ualberta.ca

Marwa.m.abolfotouh@women.asu.edu.eg

DATE OF ISSUE

30-03-2024

PES

PROOFREADING AND EDITING SERVICES

MA4130032024
