# Supplementary figures and images for: Effects of adding atracurium to Lidocaine solution during intravenous regional anesthesia in dogs
Source: Ir Vet J. 2025 Aug 6;78:16. doi: 10.1186/s13620-025-00303-5 (PMC12330004; doi:10.1186/s13620-025-00303-5)

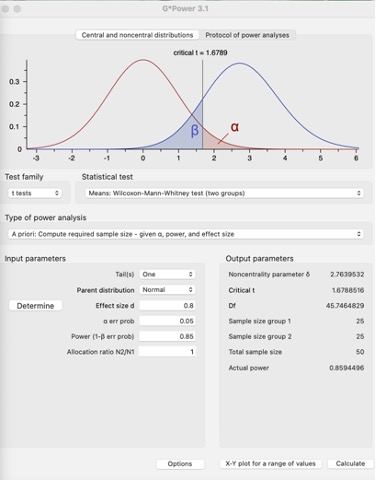

Supplement: Supplementary file 4 — Supplementary Material 4. [file 13620_2025_303_MOESM4_ESM.jpg]
