# Supplementary material for: Effects of adding atracurium to Lidocaine solution during intravenous regional anesthesia in dogs
Source: Ir Vet J. 2025 Aug 6;78:16. doi: 10.1186/s13620-025-00303-5 (PMC12330004; doi:10.1186/s13620-025-00303-5)
Supplement: Supplementary file 3 — Supplementary Material 3. [file 13620_2025_303_MOESM3_ESM.pdf]

Mansoura University
Animal Care and Use Committee
MU-ACUC

Approval Letter

Chairman

Usama Abou-Ismail

Cochairman

Irhan Abu Hashim

Members

Ahmed El-Hefnawy

Mohamed Abdraboh

Randa A. Zaghloul

Mohamed S. Hamada

Marwa Abass

Doaa Farag

Ahmed Labib

Mohamed El-Diasty

Nashwa Mo'men

Title: A Comprehensive Study of Locoregional Anesthesia Techniques with Lidocaine or Atracurium/Lidocaine Combination in Canine

Type: Independent Research.

Code number: MU-ACUC (VM.R.23.11.130)

Main Author: Asst.Prof. Marwa Abass , Department of: Surgery, Anaesthesiology and Radiology, Faculty of Veterinary Medicine, Mansoura University

Co-Authors:

1- **Co-Author:** Farid S. Ataya, Department of:
2- **Co-Author:** Aahmed M. El-Gazzar, Department of:
3- **Co-Author:** Gaber E. Batiha, Department of:
4- **Co-Author:** Alshimaa M. Farag, Department of: Internal Medicine and Infectious Disease, Faculty of Veterinary Medicine, Mansoura University

Date:29/11/2023

Dear Dr: Asst.Prof. Marwa Abass
I am pleased to let you know that your work has now been accepted by MU-ACUC.
We thank you for submitting your work to MU-ACUC and looking forward to receiving your future work.
Please do not hesitate to contact us should you have any further inquiries.

Prof. Dr. Usama Ahmed Abou-Ismail

Professor of Animal Behaviour and Welfare
Faculty of Veterinary Medicine
Chairman of Animal Care and Use Committee
Mansoura University
